# Differences in Codon Usage Bias between Photosynthesis-Related Genes and Genetic System-Related Genes of Chloroplast Genomes in Cultivated and Wild Solanum Species

**DOI:** 10.3390/ijms19103142

**Published:** 2018-10-12

**Authors:** Ruizhi Zhang, Li Zhang, Wei Wang, Zhu Zhang, Huihui Du, Zheng Qu, Xiu-Qing Li, Heng Xiang

**Affiliations:** 1 College of Pharmaceutical Sciences, Southwest University, Chongqing 400715, China; zrzxh@swu.edu.cn; 2 Department of Math and Information, China West Normal University, Nanchong, Sichuan 637000, China; albe2308@gmail.com; 3 College of Animal Science and Technology, Southwest University, Chongqing 400715, China; wcl1221@163.com (W.W.); zhangz2009swu@126.com (Z.Z.); duhuihui2010@163.com (H.D.); quzheng9509@163.com (Z.Q.); 4 Fredericton Research and Development Centre, Agriculture and Agri-Food Canada, 850 Lincoln Road, Fredericton, NB E3B 4Z7, Canada

**Keywords:** *Solanum*, codon usage bias, selective pressure, photosynthesis genes, genetic system genes

## Abstract

*Solanum* is one of the largest genera, including two important crops—potato (*Solanum tuberosum*) and tomato (*Solanum lycopersicum*). In this study we compared the chloroplast codon usage bias (CUB) among 12 *Solanum* species, between photosynthesis-related genes (Photo-genes) and genetic system-related genes (Genet-genes), and between cultivated species and wild relatives. The Photo-genes encode proteins for photosystems, the photosynthetic electron transport chain, and RuBisCO, while the Genet-genes encode proteins for ribosomal subunits, RNA polymerases, and maturases. The following findings about the *Solanum* chloroplast genome CUB were obtained: (1) the nucleotide composition, gene expression, and selective pressure are identified as the main factors affecting chloroplast CUB; (2) all these 12 chloroplast genomes prefer A/U over G/C and pyrimidines over purines at the third-base of codons; (3) Photo-genes have higher codon adaptation indexes than Genet-genes, indicative of a higher gene expression level and a stronger adaptation of Photo-genes; (4) gene function is the primary factor affecting CUB of Photo-genes but not Genet-genes; (5) Photo-genes prefer pyrimidine over purine, whereas Genet-genes favor purine over pyrimidine, at the third position of codons; (6) Photo-genes are mainly affected by the selective pressure, whereas Genet-genes are under the underlying mutational bias; (7) *S*. *tuberosum* is more similar with *Solanum commersonii* than with *Solanum bulbocastanum*; (8) *S*. *lycopersicum* is greatly different from the analyzed seven wild relatives; (9) the CUB in codons for valine, aspartic acid, and threonine are the same between the two crop species, *S. tuberosum* and *S. lycopersicum.* These findings suggest that the chloroplast CUB contributed to the differential requirement of gene expression activity and function between Photo-genes and Genet-genes and to the performance of cultivated potato and tomato.

## 1. Introduction

The genus *Solanum* is very large, containing approximately 1500 species worldwide [1,2]. This genus is very diverse and includes two very important food crops—potato (*Solanum tuberosum*) and tomato (*Solanum lycopersicum*)—as well as numerous annual or perennial plants cultivated for their ornamental flowers and fruit. *Solanum* plants are very diverse in morphology, including vines, subshrubs, shrubs, and small trees.

The complete chloroplast (plastid) genome of *S. tuberosum*, with the size of 155,296 bp, was decoded first [3], and then *Solanum bulbocastanum* and *S. lycopersicum* were decoded [4]. Comparison among these genomes detected insertions and deletions in certain coding regions or regulatory sequences and several direct and inverted repeats in the genomes. Available chloroplast complete genomes of the *Solanum* species at the time of this analysis also included eight non-tuber-bearing *Solanum* species (*S. lycopersicum* isolate TS-321, *Solanum neorickii*, *Solanum peruvianum*, *Solanum chilense*, *Solanum habrochaites*, *Solanum pimpinellifolium*, *Solanum galapagense*, and *Solanum cheesmaniae*) [5], a tuber bearing species (*Solanum commersonii*) [6], and black nightshade (*Solanum nigrum*) [7].

The genetic code is used for translation of mRNA into proteins. The standard genetic code contains 61 codons encoding 20 amino acids and 3 stop codons. The genetic code is redundant; all the amino acids, except methionine and tryptophan, can be encoded by two or more codons. Although these synonymous codons can encode the same amino acid, their usage frequency varied in different genomes [8]. The different frequencies among synonymous codons are called the codon usage bias (CUB). The CUB is widespread in organisms, and the degree of CUB is affected by nucleotide composition [9], DNA replication [10,11], translation process [12,13], tRNA abundance [14,15], gene function [16], gene length [17,18], protein structure [19,20,21], environmental temperature [22], selection at the amino acid level [23], and mutational and selective pressure [24,25]. CUB is also determined by the balance between gene mutation and natural selection [26]. Therefore, the analysis of CUB will facilitate the understanding of molecular evolution, environmental adaptation, genomic features, and gene functional requirement of the different species and of the superior agronomic performance of the cultivated species. Considering the economic significance and the species diversity of the *Solanum* genus, here we investigate whether the CUB is different among these 12 complete *Solanum* chloroplast genomes and between the two cultivated species (*S. tuberosum* and *S. lycopersicum*) and the 10 wild relative species.

Although it is known that nuclear gene CUB varies among different taxa [27], the CUB of chloroplast genomes are highly conserved and the chloroplast-encoded genes do not have the same CUB. For example, the *psbA* gene, coding the photosystem II protein D1, uses a higher frequency of the codons ending in C, whereas the overall plastid CUB favors the codons ending in A or U. This was suggested as the selective pressure that results in translation efficiency [28]. In halophytic grass *Aeluropus littoralis*, the CUB of seven genes related to photosynthesis and the respiratory system, including four nuclear genes (phosphoenolpyruvate carboxylase, NADP-malic enzyme, pyruvate orthophosphate dikinase, and glycerate kinase) and three chloroplast genes (rubisco, NADH-dehydrogenase subunit F, and cytochrome-C), has been analyzed, and the CUB is different between nucleus genes and chloroplast genes [29]. The CUB of 47 genes, classified into six categories, in the *Oncidium Gower Ramsey* chloroplast genome showed a relationship with gene function [30]. Leister classified chloroplast-encoded genes mainly into two groups: photosynthesis related and genetic system related [31]. Here, we investigated the CUB among the 12 *Solanum* chloroplast genomes because of the agricultural importance and the high degree of morphological divergence of the genus. After we found the high similarity of codon usage patterns among these 12 genomes, we compared the codon usage patterns between the photosynthesis-related (Photo-genes) and genetic system-related genes (Genet-genes), and between the cultivated crop species of potato and tomato and their wild relatives. The detected CUB differences between the two gene groups and between cultivated crops and wild relatives revealed different evolutionary mechanisms in shaping the CUB of the two functionally divergent chloroplast gene groups of the *Solanum* species and increased the understanding about how the chloroplast genomes work in the two important crops.

## 2. Materials and Methods

### 2.1. Genomes and Coding Sequences

The sequences of complete plastid genomes of twelve *Solanum* species and their coding sequences (CDS) were downloaded from GenBank (http://www.ncbi.nlm.nih.gov/genbank), which were all the available *Solanum* chloroplast genomes in GenBank at the time of this analysis. These plastid genomes were from one black nightshade species—*S. nigrum* [7]—three tuber-bearing potato species—*S. bulbocastanum* [4], *S. commersonii* [6], and *S. tuberosum* [3]—and eight tomato species—*S. neorickii* [5], *S. peruvianum* [5], *S. chilense* [5], *S. habrochaites* [5], *S. pimpinellifolium* [5], *S. galapagense* [5], *S. cheesmaniae* [5], and *S. lycopersicum* [5]. These 12 plastid genomes had 86, 85, 86, 84, 83, 83, 83, 83, 83, 83, 83 and 83 coding sequences (CDS), respectively (Table 1). CDS with a length shorter than 300 bp were excluded from CUB estimation to avoid sampling bias [32]. Thus, the final 58, 58, 58, 58, 57, 57, 57, 57, 57, 57, 57 and 57 CDS (>300 bp) were used to study the CUB for *S. nigrum*, *S. bulbocastanum*, *S. commersonii*, *S. tuberosum*, *S. neorickii*, *S. peruvianum*, *S. chilense*, *S. habrochaites*, *S. pimpinellifolium*, *S. galapagense*, *S. cheesmaniae*, and *S. lycopersicum*.

To avoid redundancy, we chose the newest reported chloroplast genome in each species if the plastid genome sequence is available from several isolates. For example, for tomato (*S. lycopersicum*) we used the isolate TS-321 (accession number KP117024) [5] but did not use the accession number NC_007898 [4]. The complete chloroplast genome of *S. pennellii* (accession number NC_024584) was removed from GenBank at the time of our analysis and therefore was not included in this study.

### 2.2. Classification of Photosynthesis Related and Genetic System Related Genes

**Figure 1:**
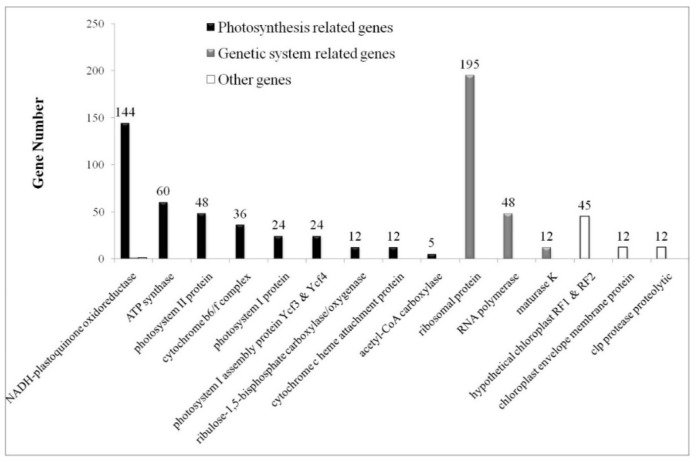
Classification of Photo-genes, Genet-genes, and Other genes for 689 *Solanum* chloroplast genes. The Photo-genes included 365 CDS, encoding proteins/subunits of NADH-plastoquinone oxidoreductase, ATP synthase, photosystem II protein, cytochrome b6/f complex, photosystem I protein, photosystem I assembly protein Ycf3 and Ycf4, ribulose-1,5-bisphosphate carboxylase/oxygenase, cytochrome c heme attachment protein, and acetyl-CoA carboxylase. The Genet-genes included 255 CDS, encoding ribosomal protein, RNA polymerase, and maturase K. The remainder 69 CDS, encoding hypothetical chloroplast RF1 and RF2, chloroplast envelope membrane protein, and clp protease proteolytic, were considered as the Other genes.

Besides analyzing and comparing the CUB of twelve *Solanum* chloroplast genomes, we classified the total 689 CDS (>300 bp) into three categories: photosynthesis related genes (Photo-genes), genetic system related genes (Genet-genes), and Other genes (Figure 1), according to their functions in the chloroplast [31]. The Photo-genes included 365 CDS, encoding proteins/subunits of NADH-plastoquinone oxidoreductase, ATP synthase, photosystem II protein, cytochrome b6/f complex, photosystem I protein, photosystem I assembly protein Ycf3 and Ycf4, ribulose-1,5-bisphosphate carboxylase/oxygenase, cytochrome c heme attachment protein, and acetyl-CoA carboxylase. The Genet-genes included 255 CDS, encoding proteins/subunits of ribosomal protein, RNA polymerase, and maturase K. The remaining 69 CDS, encoding proteins/subunits of hypothetical chloroplast RF1 and RF2, chloroplast envelope membrane protein, and clp protease proteolytic, were considered as the Other genes. The chloroplast envelope membrane protein is a probable integral membrane protein with unknown molecular function [33] and the clp protease proteolytic is an ATP-dependent protease that cleaves a number of proteins [34] but is hard to be classified into Photo- or Genet-genes. Thus, both envelope membrane proteins and clp protease proteolytic proteins were classified as the Other genes.

### 2.3. Nucleotide Composition Analyses

Our self-compiled Perl script (available on https://github.com/hxiang1019/calc_GC_content.git, January 2015) was used to compute the GC content of the entire CDS (GC_cds_) and the first (P_1_), second (P_2_), and third (P_3_) positions of codons. Because of the inequality of α and γ [35], the calculation of the nucleotide composition at the P_3_ position excluded the stop codons (UAA, UAG, and UGA) and the isoleucine codons (AUU, AUC, and AUA), and the calculation of the nucleotide composition at the P_1_, P_2_, and P_3_ positions excluded the single codons of methionine (AUG) and tryptophan (UGG). The P_12_ (the average value of P_1_ and P_2_) was used as the vertical axis while the P_3_ was used as the horizontal axis to draw the neutrality plots. The AU-bias [A_3_/(A_3_ + U_3_)] and GC-bias [G_3_/(G_3_ + C_3_)] were obtained to generate the parity rule 2 (PR2) plots, using the calculated nucleotide compositions of codons at the third position (A_3_, U_3_, C_3_ and G_3_).

### 2.4. Codon Usage Indices (CAI, ENc, GC_3s_, Gravy, and Aromo)

We used the CodonW software (John Peden, http://www.molbiol.ox.ac.uk/cu, version 1.4.2) to calculate the codon adaptation index (CAI) using *psbA*, a highly expressed plastidic gene, as the defined reference gene [28]. In order to compare with these CAI analyses, we also used CodonW’s default reference gene set (*Escherichia coli*) as the reference gene. The effective number of codons (ENc), the third synonymous codon position GC content (GC_3s_), and the gene function indices of both the amino acid hydrophobicity score (Gravy) and the aromaticity score (Aromo) for *Solanum* chloroplast genomes were also calculated using CodonW. The ENc versus GC_3s_ plots were drawn using the ENc as the vertical axis and the GC_3s_ as the horizontal axis.

### 2.5. Relative Synonymous Codon Usage and Correspondence Analyses

The CodonW was also used to calculate the relative synonymous codon usage (RSCU) of *Solanum* chloroplast genomes. Briefly, the *Solanum* chloroplast genes in the upper and lower 5% of CAI values were respectively defined as the high- and low-expression gene datasets. A statistical chi-squared test was used to compare the RSCU values between two datasets. If a codon whose frequency in the high-expression genes was significantly higher (*p* < 0.05) than in the low-expression genes, it will be classified as an optimal codon [36]. A codon with an RSCU value more than 1 (RSCU > 1) was considered as a preferred codon because its investigated usage frequency is more than the expected one. Whereas a codon with the RSCU less than 0.1 (RSCU < 0.1) was identified as a rare codon.

The RSCU values were used to carry out the correspondence analysis (COA) by comparing the variation of 59 informative codons for each *Solanum* chloroplast genome, partitioned along 59 orthogonal axes with 41 freedom degrees [37]. Statistical analyses containing the correlation analysis, significance test, and ANOVA were performed with SPSS 18.0 (http://www.spss.com/), and plots were drawn using Microsoft Excel 2007 (http://www.microsoft.com/).

## 3. Results

### 3.1. Codon Usage Indices

The mean values of the GC contents (GC_genome_, GC_cds_, GC_3s_, P_1_, P_2_, P_12_ and P_3_) and the codon indices (CAI, ENc, Gravy, and Aromo) for twelve *Solanum* chloroplast genomes were shown in Table 1. Their values show a very high similarity among twelve chloroplast genomes, although they were different plant species (black nightshade, potato, and tomato).

Regardless of whether *psbA* or *E. coli* default was used as the reference gene, the Photo-genes group had consistently higher CAI values than the Genet-genes group, with CAI differences of about 0.03 to 0.04 between the two groups (Table 2). When *psbA* was used as the reference gene, the CAI value was 0.23 for the Photo-gene group and was 0.19 for the Genet-gene group; where the CodonW’s default *E. coli* reference gene set was used as reference, the two values became 0.18 and 0.15, respectively.

The CAI value of the Other genes group was between the two CAI values of the Photo-genes and Genet-genes (Table 2). This ranking position of the Other gene group based on the CAI values may imply that the expression level of Other genes might be lower than Photo-genes but higher than Genet-genes.

The ENc index in the characterization of CUB ranged from 20 (i.e., genes with extreme bias, and only one codon per amino acid) to 61 (i.e., genes with no bias, and equally use all 61 codons). ENc can reflect the deviation degree from the equal usage of codons [38]. Generally speaking, a highly expressed gene (represented by a higher CAI value) has a greater codon bias and therefore a smaller ENc value. Thus, the relative level of gene expression can be generally estimated by CAI and ENc. For the 12 *Solanum* chloroplast genomes, their mean CAI values were 0.21 (when *psbA* was used as reference), and ENc values were greater than 48 (Table 1), indicating a low expression for most genes and a relatively weak codon adaptation across their genomes as a whole.

Nucleotide composition is an important factor influencing CUB, and the mean values of all of the *Solanum* chloroplast GC_cds_ (about 39%) were bigger than their overall genomic GC content, GC_genome_ (38%), indicating that the coding regions were likely more stable than the non-coding ones (Table 1).

**Figure 2:**
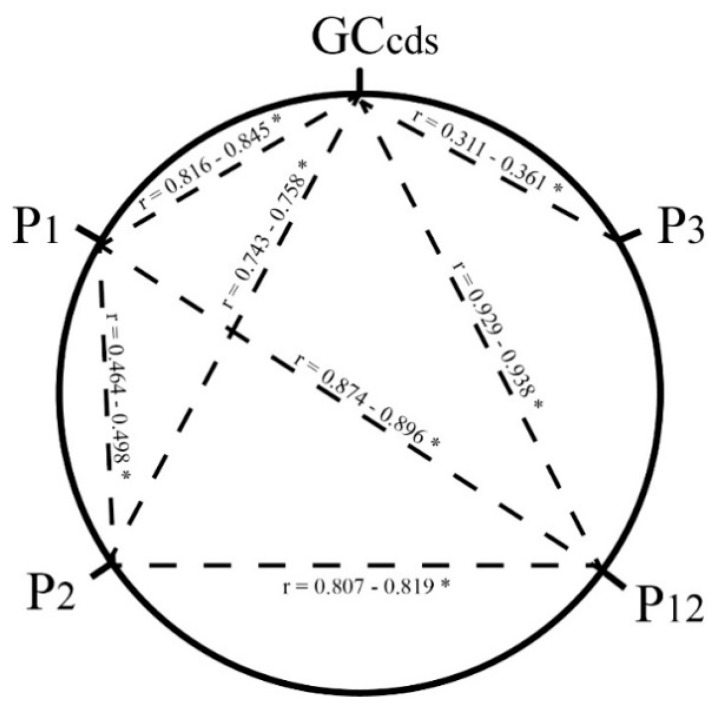
Correlation analysis among GC contents in twelve *Solanum* chloroplast genomes. The GC content of the coding sequences (GC_cds_), the first (P_1_), the second (P_2_), and the third (P_3_) codon positions, and the average value of P_1_ and P_2_ (P_12_) are shown. The dotted lines represent the significant correlation with the asterisk (* *p* < 0.01). The Spearman’s rank correlation coefficients (*r*) are 0.816–0.845 (GC_cds_ and P_1_), 0.743–0.758 (GC_cds_ and P_2_), 0.929–0.938 (GC_cds_ and P_12_), 0.311–0.361 (GC_cds_ and P_3_), 0.464–0.498 (P_1_ and P_2_), 0.874–0.896 (P_1_ and P_12_), and 0.807–0.819 (P_2_ and P_12_). The indices without lines indicate that their pairwise analysis did not detect any significant correlation. The GC content at the position P_3_ did not show a significant correlation with those at P_1_, P_2_, and P_12_, which suggests that the selective pressure affects the codon usage bias in the twelve *Solanum* chloroplast genomes.

The correlation analysis (Figure 2) shows that the average GC_cds_ was significantly correlated with not only that at the position P_1_ (*r* = 0.816–0.845, *p* < 0.01) and P_2_ (*r* = 0.743–0.758, *p* < 0.01), but also with that at the P_3_ (*r* = 0.311–0.361, *p* < 0.01) for all 12 *Solanum* chloroplast genomes, suggesting that the general GC content of chloroplast CDS were influenced by the GC content at all of the three codon positions. The correlations between P_12_ and both P_1_ (*r* = 0.874–0.896, *p* < 0.01) and P_2_ (*r* = 0.807–0.819, *p* < 0.01) were deservedly detected (Figure 2). The GC content at the position P_3_ did not show correlation with those at P_1_, P_2_, and P_12_, which suggests that natural selection affects the *Solanum* chloroplast CUB because there should be no difference among the three codon positions if there were no outside selective pressure [35].

**Figure 3:**
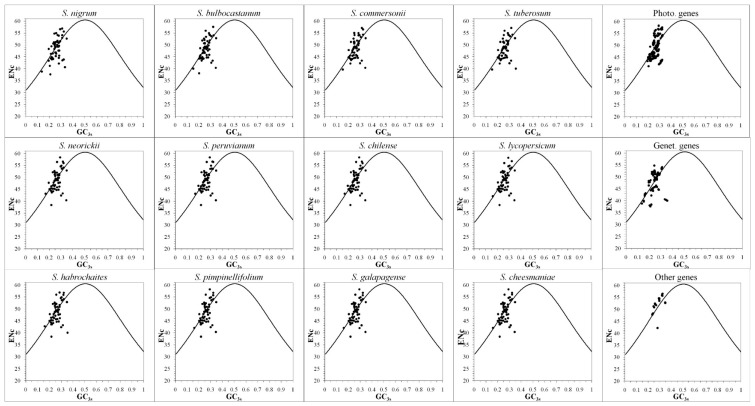
The ENc vs GC_3s_ plots of *Solanum* chloroplast genomes. The standard curve ENc = 2 + GC_3s_ + 29/[GC_3s_^2^ + (1 − GC_3s_)^2^] represents the expected ENc to GC_3s_. The dots representing most *Solanum* chloroplast genes were far away from the curve, showing that their codon usage pattern was affected by other factors besides nucleotide composition.

The ENc-plots (Figure 3) were used to investigate whether there were factors, other than the nucleotide composition, that influence CUB of *Solanum* chloroplast genomes. The CUB should be completely based on GC_3s_ and primarily determined by the nucleotide composition if genes follow the standard curve ENc = 2 + GC_3s_ + 29/[GC_3s_^2^ + (1 − GC_3s_)^2^] [38]. However, in Figure 3, the distribution of most genes did not follow the standard curve, which indicated that the nucleotide composition was not the only factor in shaping CUB and there were other factors acting on the *Solanum* chloroplast codon usage.

### 3.2. Codon Usage Biases

RSCU values, the non-uniformity in codon usage, of *Solanum* chloroplast genomes are presented in Table 3. The RSCU was calculated using the ratio that is the observed frequency divided by the expected one of a codon. A 1.00 RSCU value means no codon usage bias, a value smaller than 1.00 means a less frequent use of the codon than expected, and a value larger than 1.00 means a more frequent use of the codon than expected.

**Figure 4:**
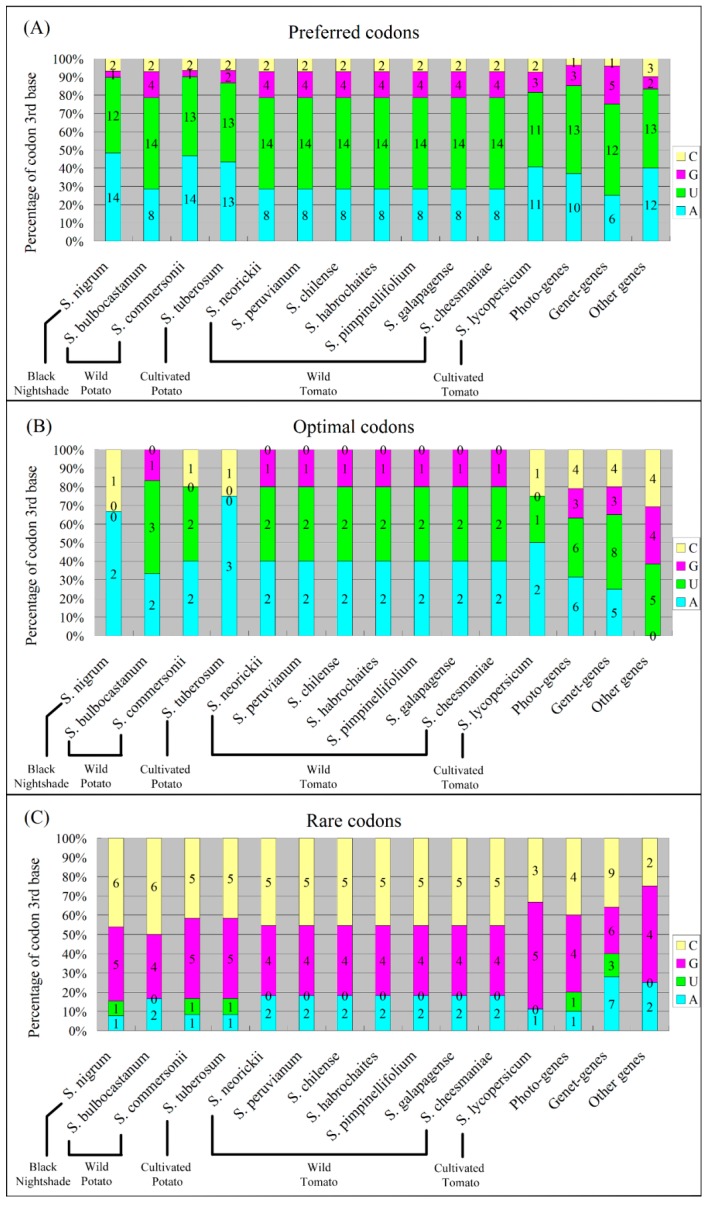
Preferred codons (A), optimal codons (B), and rare codons (C) at the third base of *Solanum* chloroplast genomes. The numbers of preferred codons (RSCU > 1), optimal codons (the usage frequency is significantly higher in high-expression genes than in low expression genes), and rare codons (RSCU < 0.1) were calculated according to Table 3 and Table S1. Cytosine (C), Guanine (G), Uracil (U), and Adenine (A) at the third base of codon were respectively shown in yellow, purple, green, and blue. For twelve *Solanum* chloroplast genomes, more than 78% preferred codons, more than 66% optimal codons, and less than 19% rare codons were A/U at the third codon position. For Photo-genes, 85% preferred codons, 63% optimal codons, and 20% rare codons ended in A or U. For Genet-genes, 75% preferred codons, 65% optimal codons, and 40% rare codons ended in A or U. For Other genes, 83% preferred codons, 38% optimal codons, and 25% rare codons ended in A or U.

The preferred codons (RSCU > 1, in bold, Table 3) in the twelve chloroplast genomes were strongly biased towards the codons ending in A or U (22–27 A/U-end in 27–30 preferred codons, more than 78%) (Figure 4A). The optimal codons (marked with * and @, Table 3) identified using chi-squared tests were similarly biased. Within the identified optimal codons, more than 66% had A/U at the third codon position (Figure 4B). More than 81% of the 9–13 rare codons (RSCU < 0.1, marked with -, Table 3) identified in twelve *Solanum* chloroplast genomes were the codons ending in G or C (Figure 4C).

In Table 3, the RSCU values between the chloroplast genomic codons of cultivated species and those of wild species were respectively compared for potatoes and tomatoes. The codon RSCU values of the cultivated potato species (*S. tuberosum*) were nearly the same with the ones of the wild potato species (*S. commersonii*), except for the two codons encoding Serine-UCA and UCG (Green Background, Table 3). There were different numbers of preferred codons between these two genomes, *S. commersonii* had 14 A-end and 1 G-end preferred codon, whereas *S. tuberosum* had 13 A-end and 2 G-end ones (Figure 4A). Although RSCU values of the other 62 codons were the same between both genomes, the codons CUU and GUU were identified as the optimal codons in wild *S. commersonii* but not in cultivated *S. tuberosum*, while the codon AUA was the optimal codon in cultivated *S. tuberosum* but not in wild *S. commersonii* (Yellow Background, Table 3), which resulted in the different optimal codon numbers between the chloroplast genes of the cultivated potato species *S. tuberosum* and those of the wild potato species *S. commersonii* (Figure 4B).

All of the seven wild tomatoes (*S. neorickii*, *S. peruvianum*, *S. chilense*, *S. habrochaites*, *S. pimpinellifolium*, *S. galapagense*, and *S. cheesmaniae*) had the same RSCU values (Table 3), and the same preferred codons, optimal codons, and rare codons (Figure 4). However, a number of differences between the seven wild tomatoes and the cultivated tomato species (*S. lycopersicum*) were found (Blue Background, Table 3). For example, for encoding valine, *S. lycopersicum* preferred to use GUU, whereas seven wild tomatoes liked GUG, but GUG was the rare codon in the *S. lycopersicum* chloroplast genome; for encoding aspartic acid, *S. lycopersicum* preferred to use GAC, whereas seven wild tomatoes preferred GAU; for encoding serine, *S. lycopersicum* preferred to use UCA, whereas seven wild tomatoes preferred UCU; for encoding threonine, *S. lycopersicum* preferred to use ACA whereas seven wild tomatoes preferred ACU and ACC. These differences feature the divergence of preferred codons, optimal codons, and rare codons between the chloroplast genomes of seven wild tomatoes and that of the cultivated tomato (Figure 4).

Within the divergent codon bias patterns between seven wild tomatoes and cultivated tomato species (Blue Background, Table 3), several patterns were similar between cultivated tomato *S. lycopersicum* and the cultivated potato *S. tuberosum* (underlined, Table 3). For example, for encoding valine, both *S. lycopersicum* and *S. tuberosum* preferred to use GUU, not GUG; for encoding aspartic acid, both *S. lycopersicum* and *S. tuberosum* preferred to use GAC, not GAU; for encoding threonine, both *S. lycopersicum* and *S. tuberosum* preferred to use ACA, but did not prefer ACU and ACC. Thus, although there were some differences between tomato *S. lycopersicum* and potato *S. tuberosum*, several codons were preferred by cultivated species but not preferred by wild species.

The preferred codons, optimal codons, and rare codons for the three gene categories (Photo-, Genet-, and Other-genes) were also identified using their RSCU values (Table S1). Their bias patterns were similar for the twelve genomes, i.e., they prefer using the codons with A/U at the third position. There were 23 (85.19% of a total 27), 18 (75.00% of a total 24), and 25 (83.33% of a total 30) preferred codons ending in A or U, for Photo-genes, Genet-genes, and Other genes, respectively (Figure 4A). Most rare codons had a G/C-end for the three groups of genes (Figure 4C).

### 3.3. Parity Rule 2 Plot Analyses

**Figure 5:**
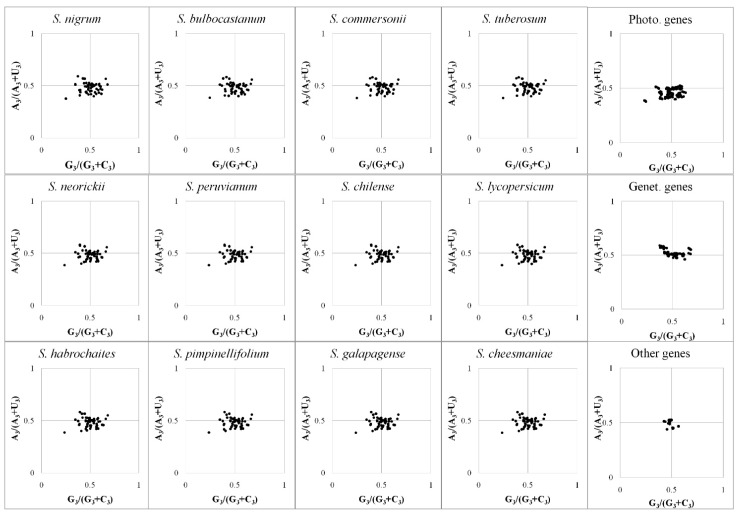
The PR2-bias plots of *Solanum* chloroplast genomes. Genes were plotted based on their GC bias [G_3_/(G_3_ + C_3_)] and AU bias [A_3_/(A_3_ + U_3_)] in the third codon position. The mean GC-biases of *S. nigrum*, *S. bulbocastanum*, *S. commersonii*, *S. tuberosum*, *S. neorickii*, *S. peruvianum*, *S. chilense*, *S. lycopersicum*, *S. habrochaites*, *S. pimpinellifolium*, *S. galapagense*, *S. cheesmaniae*, Photo-genes, Genet-genes, and Other genes were 0.497, 0.498, 0.499, 0.498, 0.497, 0.498, 0.497, 0.497, 0.497, 0.496, 0.497, 0.497, 0.482, 0.515, and 0.513, respectively; while their mean AU-biases were 0.484, 0.484, 0.483, 0.484, 0.486, 0.486, 0.486, 0.485, 0.486, 0.486, 0.486, 0.486, 0.461, 0.519, and 0.487, respectively. The GC bias of 0.482 and AU bias of 0.461 showed a stronger preference of pyrimidines over purines for Photo-genes, whereas the GC bias of 0.515 and AU bias of 0.519 showed a preference of purines over pyrimidines for Genet-genes.

Because of the bias between pyrimidine and purine for both the preferred and the optimal codons of the twelve *Solanum* chloroplast genomes (Figure 4), all codons were examined using PR2 plot analysis (Figure 5). In the PR2 plot, both the mean GC-biases [G_3_/(G_3_ + C_3_)] and the mean AU-biases [A_3_/(A_3_ + U_3_)] of the 12 *Solanum* species were smaller than 0.5 (Figure 5). Thus, for all of the 12 *Solanum* chloroplast genomes, pyrimidine was preferred over purine (U over A, and C over G), at the third position (Figure 5). However, the PR2 plot demonstrated the preference of pyrimidine over purine in Photo-genes, while purine was preferred over pyrimidine in Genet-genes (Figure 5).

In a gene where CUB is only influenced by nucleotide composition, the third positions should have the identical distribution between G_3_ and C_3_, as well as A_3_ and U_3_, and this intra-strand rule is theoretically respected regardless of the G + C content [39,40]. Thus, the asymmetry between pyrimidines and purines suggests that there were other factors besides nucleotide composition affecting the *Solanum* chloroplast CUB.

### 3.4. Neutrality Plot Analyses

**Figure 6:**
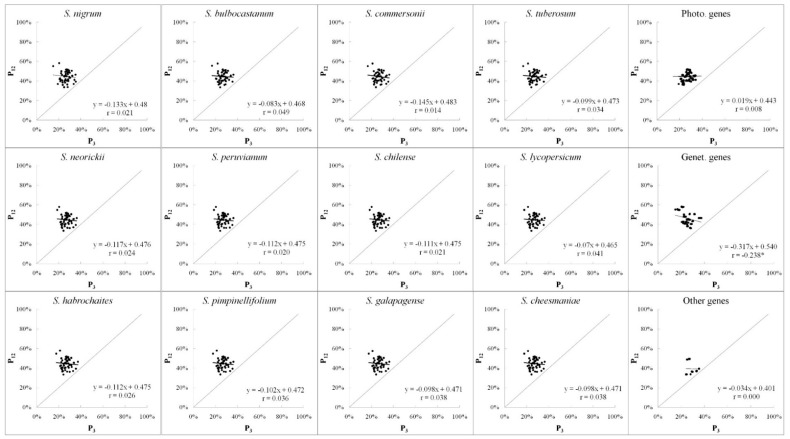
Neutrality plots of *Solanum* chloroplast genomes. Individual genes were plotted based on the mean GC content in the first and second codon position (P_12_) versus the GC content of the third codon position (P_3_). Regression lines and Spearman’s rank correlation coefficients (r) are shown, with the asterisk (*) denoting significance at the *p* < 0.01 level. Note that both the nearly horizontal regression curve and small correlation coefficient (*r* = 0.008, *p* > 0.05) present a strong selective pressure for the photosynthesis related genes in *Solanum* chloroplast genomes. Whereas for the Genet-genes, its correlation coefficient of 0.238 with the significant correlation (*p* < 0.01, marked with *) indicated that the mutational bias plays a more active role than the selective pressure in CUB for the *Solanum* chloroplast genetic system related genes.

The neutrality plot analysis (Figure 6) was carried out to characterize the correlation between P_12_ and P_3_ positions, and then identify the effects of both selection and mutation on CUB. The regression slopes and correlation coefficients (Figure 6) did not demonstrate a significant correlation between P_12_ and P_3_ positions (Figure 2) in the twelve chloroplast genomes. The low relative neutralities (all values < 14.5%) in the twelve chloroplast genomes suggested that natural selection played an important role in shaping the CUB of the *Solanum* chloroplast genomes.

The nearly horizontal regression curve (very low relative neutrality value of 1.9%) and nonsignificant correlation (*r* = 0.008, *p* > 0.05) between P_12_ and P_3_ for the Photo-genes presented a strong selective pressure for the *Solanum* chloroplast photosynthesis-related genes (Figure 6). Whereas, for the Genet-genes, its correlation coefficient of 0.238 (*p* < 0.01, marked with * in Figure 6) and high relative neutrality value of 31.7% indicated that the underlying mutational bias played a more active role than the selective pressure in CUB for the *Solanum* chloroplast genetic system related genes.

### 3.5. Correspondence Analyses

The nucleotide composition was not the only factor shaping the *Solanum* chloroplast CUB according to both ENc-plot and PR2 plot analyses. Therefore, we used the correspondence analysis (COA) [37], a multivariate statistical method, to investigate other factors affecting CUB. The COA can describe the distribution of genes and their corresponding codons, unveiling potential influences on CUB [20].

The COA generated a series of orthogonal axes that represent the factors relevant to CUB, and Axis 1 and Axis 2 as shown in Figure S1. The combined values of the first four axes were shown in the legends of Figure S1. For the 12 species, the first axis represented the primary factor because the 9.56–9.96% variation of Axis 1 was greater than the similar variations of the three other axes (7.60–7.93% variation of Axis 2, 7.28–7.54% variation of Axis 3, and 6.78–7.03% variation of Axis 4). The Axis 1 values were found to be significantly correlated with GC_3s_, ENc, and CAI when all genes were analyzed together in most of the twelve *Solanum* chloroplast genomes (Table S2). These correlations indicated that the nucleotide composition (represented by GC_3s_), the codon usage evenness (represented by ENc), and the gene expression level (represented by CAI) may play the main roles in determining the CUB of the *Solanum* chloroplast genomes. The third axis was significantly correlated with Length_cds_ (*p* < 0.05) (Table S2), suggesting that the gene lengths were also responsible, to a certain degree, for the codon usage variation of the twelve *Solanum* chloroplast genomes.

The COA generated Axis 1 (The primary factor) of Photo-genes and Genet-genes were significantly correlated with GC_cds_ (*p* < 0.01) (Table 4), showing that nucleotide composition played an important role in determining the CUB of these two functionally different genes. However, GC_3s_ was significantly correlated with Axis 1 of the Photo-genes (correlation coefficient of 0.629) and Axis 2 of the Genet-genes (correlation coefficient of 0.589), but not Axis 1 of Genet-genes (correlation coefficient of −0.030, *p* > 0.05) (Table 4), which indicates that nucleotide composition at the third position was the primary factor for the CUB of Photo-genes but the secondary factor for Genet-genes. Moreover, both the gene function (represented by Gravy and Aromo) and the gene length (represented by Length_cds_) were significantly correlated with Axis 1 of the Photo-genes (*p* < 0.01) but was not correlated with that of the Genet-genes (*p* > 0.05) (Table 4), supporting the functional importance of photosynthesis related-genes in plant chloroplasts.

## 4. Discussion

### 4.1. Similarity in Codon Usage Among the Twelve Solanum Chloroplast Genomes

As the first and second most commonly grown vegetable crops in the world, potato and tomato are economically important. Here, the CUB of the complete chloroplast genomes for twelve *Solanum* species (containing one black nightshade, three potatoes and eight tomatoes) were investigated. CUB, an important feature of species, can reflect genome evolution and expression patterns and has been reported in great numbers of organisms [41]. The highly similar codon usage patterns among the twelve chloroplast genomes in this study supports the previous reports that the chloroplast genome among land plants generally has a highly conserved structure [42,43,44] from the codon usage aspect.

This study determined both optimal and preferred codons in *Solanum* chloroplast genomes using RSCU values, although the predicted effects of CUB on gene expression were likely to be not very great according to CAI and ENc (Table 1). Most of the optimal and the preferred codons in *Solanum* chloroplast genomes ended in A or U (Figure 4). This A/U-end bias of the *Solanum* chloroplast genomes might be consistent with its genomic low-GC content, particularly its low-GC content at third codon position (P_3_) with the mean of about 27% (Table 1). It is similar to the pattern of most organisms, namely G/C-rich species tend to possess G/C-rich optimal codons while their A/U-rich counterpart prefers A/U-rich optimal codons [45], thus revealing that one of the most important factors in determining *Solanum* chloroplast CUB is nucleotide composition. This result was supported by the COA (Figure S1) and its correlation analyses (Table S2), where Axis 1 (the primary factor representative) was found to be significantly correlated with GC_3s_ (the nucleotide composition representative) in most *Solanum* chloroplast genomes. The A/U-end bias has been observed in both chloroplast genomes [28,46,47,48] and bacteria [49], thus supporting the origin of chloroplasts from *Cyanobacteria* by endosymbiosis [50]. Additionally, previous studies have shown that codons ending in A or U were preferentially used in dicot genomic genes [51,52]. Further evidence of this point has been provided by the present study, the genome wide analysis of the chloroplast genomes of 12 *Solanum* species, belonging to the dicot plants.

### 4.2. Impact of Selective Pressure on Shaping CUB in the Solanum Chloroplast Genomes

In 1950, Erwin Chargaff found that the bases’ abundance of guanine (G) is equal to cytosine (C) and adenine (A) is equal to thymine (T) in DNA, which was referred to as Chargaff’s rule or parity rule 1 [53]. It was later demonstrated that both equalities should be observed at the state of equilibrium, when there is no mutation and selection pressures in any of the two DNA strands, referred to as parity rule 2 (PR2) [40]. In the present PR2 analysis, codons in twelve *Solanum* chloroplast genomes were found to prefer pyrimidines over purines at the third position, i.e., the base U was used more frequently than A, and C was used more frequently than G (Figure 5). Thus, although nucleotide composition is considered as the main factor responsible for *Solanum* chloroplast CUB, the asymmetry between pyrimidines and purines demonstrates that some other factors besides nucleotide composition also affect the CUB of *Solanum* chloroplast genomes [39]. In the ENc-plots (Figure 3), the distribution of most genes did not follow the standard curve, which provided an additional support to this opinion. Within the factors, the mutation and the selective pressure are the most plausible reasons to introduce this asymmetrical usage between pyrimidines and purines in the DNA sequence [54,55,56].

Therefore, the neutrality plot analysis for the *Solanum* chloroplast CUB was executed to characterize the roles of mutation and selective pressure. In the neutrality plot (Figure 6), the regression slopes and correlation coefficients did not demonstrate a significant correlation between P_12_ and P_3_ in the twelve *Solanum* chloroplast genomes, which suggests that the natural selection pressure took an active role in the CUB of *Solanum* chloroplast genomes. This pattern was detected in the other CUB analyses of different plant chloroplast genomes [52,57,58,59], particularly in 103 plastid genomes [28]. Thus, the natural selection pressure was one of the main factors shaping the CUB of plant chloroplasts. This selective pressure on CUB was argued to increase translational efficiency for plastid genomes [28]. In this study, the low relative neutralities (all values < 14.5%) in the twelve genomes confirmed that selective pressure shaped the CUB in the *Solanum* chloroplast genomes.

### 4.3. Different RSCU Values between Cultivated Species and Wild Species

The RSCU values of chloroplast genomic codons of the cultivated potato species (*S. tuberosum*) were highly similar with that of the wild potato species *S. commersonii* but different from that of the other wild potato species *S. bulbocastanum* (Table 3), suggesting that the chloroplast genome of *S. tuberosum* has a closer relationship with that of *S. commersonii* than with that of *S. bulbocastanum*, which is in agreement with the similar relationship at the genome sequence cluster level [60].

Although seven wild tomatoes (*S. neorickii*, *S. peruvianum*, *S. chilense*, *S. habrochaites*, *S. pimpinellifolium*, *S. galapagense*, and *S. cheesmaniae*) came from a variety of worldwide regions, all of them had the exact same RSCU values (Table 3) and same preferred codons, optimal codons, and rare codons (Figure 4) in their chloroplast genomes. Therefore, we speculate that all of them probably originated from a common ancestor. The notable divergence of codon RSCU values of chloroplast genomes between seven wild tomato species and the cultivated tomato species (*S. lycopersicum*) was identified (Table 3). There were differences in preferred codons, optimal codons, and rare codons between both the cultivated and the wild tomato chloroplast genomes (Figure 4). Moreover, a few codons were preferred by both the cultivated tomato (*S. lycopersicum*) and potato (*S. tuberosum*), but not preferred by seven wild tomatoes species (Table 3), indicative of an important role of artificial selection on codon usage in the plant chloroplast genomes during crop domestication and evolution.

### 4.4. Differences between Photo-Genes and Genet-Genes

Although it is known that the CUB of plant chloroplast genomes tend to have an A/U bias and are mainly affected by the factors of nucleotide composition and selective pressure [28,47], the present study compared the CUB differences between photosynthesis-related genes and genetic system-related genes of chloroplast genomes. We found that: (1) Photo-genes always had higher CAI values than Genet-genes (Table 2). (2) The gene function (represented by Gravy and Aromo) was significantly correlated with the COA-generated Axis 1 of Photo-genes (*p* < 0.01) but was not correlated with that of Genet-genes (*p* > 0.05) (Table 4). (3) In the PR2 plot (Figure 5), Photo-genes preferred pyrimidine over purine, whereas Genet-genes preferred purine over pyrimidine, at the third position of codon. (4) In the neutrality plot (Figure 6), Photo-genes were mainly affected by the selective pressure; whereas, Genet-genes were under the underlying mutational bias.

As one of the most widely used indices for analyzing CUB, CAI measures the degree of a given gene in using high fitness codons with respect to a reference set of genes; therefore, it is a measure of adaptation. If a high expression gene, such as the photo-regulated *psbA*, was used as the reference, a higher CAI value with the range between 0 and 1 indicates a potentially higher gene expression [61]. The Photo-genes group of the *Solanum* chloroplast genomes consistently had higher CAI values than the Genet-genes group, regardless of whether the *psbA* gene or the *E. coli* default was used as the reference (Table 2). These results may support the idea that Photo-genes have a higher expression level than Genet-genes. The fast growth of plants requires a high expression of photosynthesis-related genes. Highly-expressed genes may need to choose suitable codons in translation for the efficient use of resources within the cell in competition with other genes [62]. A higher CAI also indicates a stronger adaptation [61], which strongly demonstrates natural selection. For instance, the H3N2 canine influenza virus has a higher CAI value in chickens than in canines, felines, and humans because the virus originated from avian species and was then transmitted to mammals [63]. Thus, Photo-genes may have a higher CUB adaptation for the requirement of high gene expression than the Genet-genes in the *Solanum* chloroplast genomes.

In the correlation analyses of the COA axes (Table 4), the gene function (represented by Gravy and Aromo) was significantly correlated with the primary factor (represented by Axis 1) of Photo-genes, but was the tertiary factor (represented by Axis 3) of Genet-genes, strongly suggesting that photosynthesis is critically important for plants, which plays the primary role in shaping the CUB of the *Solanum* chloroplast. In plants, the chloroplast genome copy number increased significantly after leaves become green and functional in plants, such as in maize [64]. Chloroplasts likely also interact with mitochondria at the level of genome copy per cell [64]. Organelle DNA stoichiometric variation is known to affect plant growth speed and pollen fertility [65], further indicating the importance of organelle gene function for plant growth and development. Chloroplast RNA is known to have editing [66], likely for a better adaptation in resources for a more effective translation and an improved performance of gene function. Thus, to meet the functions required by plant photosynthesis, chloroplast genomes may not only vary its copy number per cell, expression activity, or RNA editing, but also more selectively use adaptive codons of Photo-genes than other genes.

Any deviation from PR2 will result in an asymmetric nucleotide composition [40]. This asymmetric phenomenon was previously found between the leading strand and the lagging strand of bacteria, i.e., the leading strand prefers G over C and T over A, but vice versa for the lagging strand [67]. There are two main explanations with regard to the different bias in nucleotide composition of these two DNA strands. The first one suggests that the different mutational pressure between the leading and the lagging strands during replication and transcription should contribute for their asymmetric nucleotide composition [54]. The second one considers that the mechanism of nucleotide composition asymmetry results from different selective pressure between these two strands [55]. Similar to this phenomenon of the leading and lagging strand, the different nucleotide composition asymmetry between Photo-genes and Genet-genes was detected in our PR2 plot analysis of *Solanum* chloroplast genomes (Figure 5). In consideration of our neutrality plot analysis, in which Photo-genes were mainly affected by the selective pressure, while Genet-genes were under the mutational pressure (Figure 6), we speculate that the preference of pyrimidines over purines in Photo-genes was mainly generated by its selective pressure, while the preference of purines over pyrimidines in Genet-genes was mainly generated by its mutational pressure. Of course, although both mutational and selective pressures can independently introduce the nucleotide composition asymmetry, the combination and cumulative effect of both mechanisms might be the most plausible explanation for the asymmetry [56]. Thus, further investigation will be required to understand the biology under this finding.

According to the genetic codon table, the mutations of DNA sequences occurring in the third position usually are synonymous (encoded amino acid is not changed), while the ones in the first or the second positions usually are non-synonymous (encoded amino acid is changed) [68,69]. As the non-synonymous mutations result in an amino acid change then in a biological change of the organism, they are subject to natural selection. This means that if there is no outside selective pressure, mutations should occur randomly rather than in a certain direction under the condition of pressure, and therefore there should be no difference among the base preferences at the three codon positions [35]. In our neutrality plot (Figure 6), the significant correlation (no difference) between P_12_ and P_3_ of the Genet-genes indicates that the random and underlying mutational bias could take an active role for the *Solanum* chloroplast genetic system related genes, whereas the difference (no significant correlation) between P_12_ and P_3_ of the Photo-genes presents a strong selective pressure for the *Solanum* chloroplast photosynthesis related genes. Therefore, we speculate that the Photo-genes in plant species were selected to adapt to the intracellular environment, such as substrate availability, and to achieve a high translation efficiency due to its functional importance for the plants, particularly for high performance crops, to survive in natural and artificial selections.

## Figures and Tables

**Table 1:** Genomic features of twelve *Solanum* chloroplast genomes.

	Black Nightshade	Potato			Tomato							
	Wild Species			Cultivated Species	Wild Species							Cultivated Species
	*S. nigrum*	*S. bulbocastanum*	*S. commersonii*	*S. tuberosum*	*S. neorickii*	*S. peruvianum*	*S. chilense*	*S. habrochaites*	*S. pimpinellifolium*	*S. galapagense*	*S. cheesmaniae*	*S. lycopersicum*
**Accession No.**	KM489055	NC_007943	KM489054	NC_008096	KP117025	KP117026	KP117021	KP117023	KP117027	KP117022	KP117020	KP117024
**Genomic size (bp)**	155,432	155,371	155,525	155,296	155,513	155,561	155,528	155,465	155,442	155,474	155,520	155,452
**CDS**	86	85	86	84	83	83	83	83	83	83	83	83
**CDS_>300bp_**	58	58	58	58	57	57	57	57	57	57	57	57
**Length_cds_ (aa)**	431.22 ± 475.46	426.60 ± 476.99	430.71 ± 475.30	426.38 ± 476.96	429.84 ± 479.54	429.96 ± 479.53	429.84 ± 479.54	429.84 ± 479.54	429.84 ± 479.54	429.84 ± 479.54	429.89 ± 479.54	429.76 ± 477.10
**GC_genome_**	0.38	0.38	0.38	0.38	0.38	0.38	0.38	0.38	0.38	0.38	0.38	0.38
**GC_cds_**	0.39 ± 0.04	0.39 ± 0.04	0.39 ± 0.04	0.39 ± 0.04	0.39 ± 0.03	0.39 ± 0.03	0.39 ± 0.03	0.39 ± 0.04	0.39 ± 0.04	0.39 ± 0.03	0.39 ± 0.03	0.39 ± 0.04
**P_1_**	0.49 ± 0.06	0.49 ± 0.06	0.49 ± 0.06	0.49 ± 0.06	0.49 ± 0.06	0.49 ± 0.06	0.49 ± 0.06	0.49 ± 0.06	0.49 ± 0.06	0.49 ± 0.06	0.49 ± 0.06	0.49 ± 0.06
**P_2_**	0.40 ± 0.06	0.40 ± 0.06	0.40 ± 0.06	0.40 ± 0.06	0.40 ± 0.06	0.40 ± 0.06	0.40 ± 0.06	0.40 ± 0.06	0.40 ± 0.06	0.40 ± 0.06	0.40 ± 0.06	0.40 ± 0.06
**P_12_**	0.44 ± 0.05	0.45 ± 0.05	0.44 ± 0.05	0.45 ± 0.05	0.45 ± 0.05	0.45 ± 0.05	0.45 ± 0.05	0.45 ± 0.05	0.45 ± 0.05	0.45 ± 0.05	0.45 ± 0.05	0.45 ± 0.05
**P_3_**	0.27 ± 0.04	0.27 ± 0.04	0.27 ± 0.04	0.27 ± 0.04	0.27 ± 0.04	0.27 ± 0.04	0.27 ± 0.04	0.27 ± 0.04	0.27 ± 0.04	0.27 ± 0.04	0.27 ± 0.04	0.27 ± 0.04
**CAI** (ref. *psbA*)	0.21 ± 0.04	0.21 ± 0.04	0.21 ± 0.04	0.21 ± 0.04	0.21 ± 0.04	0.21 ± 0.04	0.21 ± 0.04	0.21 ± 0.04	0.21 ± 0.04	0.21 ± 0.04	0.21 ± 0.04	0.21 ± 0.04
**CAI** (ref. *E. coli*)	0.17 ± 0.04	0.17 ± 0.04	0.17 ± 0.04	0.17 ± 0.04	0.17 ± 0.04	0.17 ± 0.04	0.17 ± 0.04	0.17 ± 0.04	0.17 ± 0.04	0.17 ± 0.04	0.17 ± 0.04	0.17 ± 0.04
**ENc**	48.54 ± 4.44	48.44 ± 4.23	48.63 ± 4.18	48.46 ± 4.14	48.69 ± 4.32	48.66 ± 4.31	48.65 ± 4.30	48.69 ± 4.29	48.70 ± 4.35	48.70 ± 4.36	48.70 ± 4.36	48.56 ± 4.23
**Gravy**	−0.06 ± 0.50	−0.06 ± 0.50	−0.05 ± 0.50	−0.06 ± 0.50	−0.05 ± 0.50	−0.05 ± 0.50	−0.05 ± 0.50	−0.05 ± 0.50	−0.05 ± 0.50	−0.05 ± 0.50	−0.05 ± 0.50	−0.06 ± 0.50
**Aromo**	0.10 ± 0.04	0.10 ± 0.04	0.10 ± 0.04	0.10 ± 0.04	0.10 ± 0.04	0.10 ± 0.04	0.10 ± 0.04	0.10 ± 0.04	0.10 ± 0.04	0.10 ± 0.04	0.10 ± 0.04	0.10 ± 0.04

CDS_>300bp_: Number of the coding sequences with a length longer than 300 bp, which was used in the study. Length_cds_: length of the coding sequences (>300 bp). GC_genome_: GC content of the whole genome. GC_cds_: GC content of the coding sequences (>300 bp). P_1_, P_2_, and P_3_: GC content at the first, second, and third codon position, respectively. P_12_: average value of P_1_ and P_2_. CAI: codon adaptation index. *psbA*: chloroplast photosystem II protein D1. ENc: effective number of codons. Gravy: amino acid hydrophobicity. Aromo: amino acid aromaticity.

**Table 2:** Features of three gene categories (Photo-, Genet-, and Other-genes) of *Solanum* chloroplast genomes.

	Photo-Genes	Genet-Genes	Other Genes
CDS	581	353	75
CDS_>300bp_	365	255	69
Length_cds_ (aa)	355.23 ± 187.59	314.09 ± 331.29	1246.13 ± 960.28
GC_cds_	0.39 ± 0.03	0.39 ± 0.03	0.37 ± 0.03
P_1_	0.50 ± 0.06	0.48 ± 0.06	0.46 ± 0.07
P_2_	0.39 ± 0.04	0.43 ± 0.07	0.33 ± 0.03
P_12_	0.45 ± 0.04	0.46 ± 0.05	0.39 ± 0.05
P_3_	0.27 ± 0.03	0.26 ± 0.04	0.31 ± 0.04
CAI (ref. *psbA*)	0.23 ± 0.05	0.19 ± 0.03	0.20 ± 0.03
CAI (ref. *E. coli*)	0.18 ± 0.04	0.15 ± 0.03	0.17 ± 0.02
ENc	48.65 ± 4.08	47.57 ± 4.30	52.31 ± 2.75
Gravy	0.26 ± 0.44	−0.46 ± 0.21	−0.20 ± 0.33
Aromo	0.12 ± 0.04	0.07 ± 0.03	0.12 ± 0.01

CDS_>300bp_: Number of the coding sequences with the length bigger than 300 bp, which was used in the study. Length_cds_: length of the coding sequences (>300 bp). GC_cds_: GC content of the coding sequences (>300 bp). P_1_, P_2_, and P_3_: GC content at the first, second, and third codon position, respectively. P_12_: average value of P_1_ and P_2_. CAI: codon adaptation index. *psbA*: chloroplast photosystem II protein D1. ref.: reference gene set. ENc: effective number of codons. Gravy: amino acid hydrophobicity. Aromo: amino acid aromaticity.

**Table 3:** The relative synonymous codon usage (RSCU) values of twelve *Solanum* chloroplast genomes. The optimal codons, preferred codons and rare codons were identified by the RSCU values. The optimal codon, marked with * (*p* < 0.01) and @ (*p* < 0.05) was defined as a codon whose usage frequency in the high-expression genes was significantly higher than in the low-expression genes by the chi-squared contingency test. The preferred codon (shown in bold) was a codon with the RSCU value more than 1 (RSCU > 1), whose investigated usage frequency is more than the expected one. Whereas the rare codon (marked with -) was a codon with the RSCU less than 0.1 (RSCU < 0.1), representing a very low usage frequency. Green background: There were only two different RSCU values between the wild *S. commersonii* and the cultivated *S. tuberosum*; while others were the same between the chloroplast genomes. Yellow background: Within the same RSCU values between the wild *S. commersonii* and the cultivated *S. tuberosum*, the different optimal codons were identified using the same chi-squared test. Blue background: The notable differences between the cultivated *S. lycopersicum* and seven wild tomatoes. RSCU values underlined: The preferred codon patterns of the cultivated tomato *S. lycopersicum* were different from those of the seven wild tomatoes, but similar with those of cultivated potato *S. tuberosum*.

Amino Acid	Codon	Black Nightshade	Potato			Tomato							
			Wild Species		Cultivated Species	Wild Species							Cultivated Species
		*S. nigrum*	*S. bulbocastanum*	*S. commersonii*	*S. tuberosum*	*S. neorickii*	*S. peruvianum*	*S. chilense*	*S. habrochaites*	*S. pimpinellifolium*	*S. galapagense*	*S. cheesmaniae*	*S. lycopersicum*
Phe	UUU	1.00	**1.78**	1.00	1.00	**1.78**	**1.78**	**1.78**	**1.78**	**1.78**	**1.78**	**1.78**	**1.78**
	UUC	1.00	0.22	1.00	1.00	0.22	0.22	0.22	0.22	0.22	0.22	0.22	0.22
Leu	UUA	0.33	**2.50@**	0.35	0.35	**2.50@**	**2.50@**	**2.50@**	**2.50@**	**2.50@**	**2.50@**	**2.50@**	0.75
	UUG	**2.00**	**1.50**	**2.12**	**2.12**	**1.50**	**1.50**	**1.50**	**1.50**	**1.50**	**1.50**	**1.50**	**2.75**
	CUU	**2.33**	1.00	**2.12@**	**2.12**	1.00	1.00	1.00	1.00	1.00	1.00	1.00	**1.50**
	CUC	0.00-	0.25	0.00-	0.00-	0.25	0.25	0.25	0.25	0.25	0.25	0.25	0.25
	CUA	**1.33**	0.50	**1.41**	**1.41**	0.50	0.50	0.50	0.50	0.50	0.50	0.50	0.50
	CUG	0.00-	0.25	0.00-	0.00-	0.25	0.25	0.25	0.25	0.25	0.25	0.25	0.25
Ile	AUU	**1.31**	**1.66**	**1.41**	**1.41**	**1.61**	**1.61**	**1.61**	**1.61**	**1.61**	**1.61**	**1.61**	**1.58**
	AUC	0.00-	0.41	0.00-	0.00-	0.43	0.43	0.43	0.43	0.43	0.43	0.43	0.79
	AUA	**1.69**	0.93	**1.59**	**1.59@**	0.96	0.96	0.96	0.96	0.96	0.96	0.96	0.63
Met	AUG	1.00	1.00	1.00	1.00	1.00	1.00	1.00	1.00	1.00	1.00	1.00	1.00
Val	GUU	**2.50**	**1.33**	**2.50@**	**2.50**	**1.71**	**1.71**	**1.71**	**1.71**	**1.71**	**1.71**	**1.71**	3.11 *
	GUC	0.00-	0.00-	0.00-	0.00-	0.00-	0.00-	0.00-	0.00-	0.00-	0.00-	0.00-	0.00-
	GUA	**1.50**	0.67	**1.50**	**1.50**	0.57	0.57	0.57	0.57	0.57	0.57	0.57	0.89
	GUG	0.00-	**2.00@**	0.00-	0.00-	**1.71@**	**1.71@**	**1.71@**	**1.71@**	**1.71@**	**1.71@**	**1.71@**	0.00-
Tyr	UAU	**2.00**	**2.00**	**2.00**	**2.00**	**1.67**	**1.67**	**1.67**	**1.67**	**1.67**	**1.67**	**1.67**	**1.67**
	UAC	0.00-	0.00-	0.00-	0.00-	0.33	0.33	0.33	0.33	0.33	0.33	0.33	0.33
STOP	UAA	**3.00**	**1.50**	**3.00**	**3.00**	**1.50**	**1.50**	**1.50**	**1.50**	**1.50**	**1.50**	**1.50**	**3.00**
	UAG	0.00-	**1.50**	0.00-	0.00-	**1.50**	**1.50**	**1.50**	**1.50**	**1.50**	**1.50**	**1.50**	0.00-
	UGA	0.00-	0.00-	0.00-	0.00-	0.00-	0.00-	0.00-	0.00-	0.00-	0.00-	0.00-	0.00-
His	CAU	**1.60**	**2.00**	**1.67**	**1.67**	**2.00**	**2.00**	**2.00**	**2.00**	**2.00**	**2.00**	**2.00**	**1.60**
	CAC	0.40	0.00-	0.33	0.33	0.00-	0.00-	0.00-	0.00-	0.00-	0.00-	0.00-	0.40
Gln	CAA	**1.25**	**2.00**	**1.25**	**1.25**	**2.00**	**2.00**	**2.00**	**2.00**	**2.00**	**2.00**	**2.00**	**1.33**
	CAG	0.75	0.00-	0.75	0.75	0.00-	0.00-	0.00-	0.00-	0.00-	0.00-	0.00-	0.67
Asn	AAU	**1.60**	**1.60**	**1.60**	**1.60**	**1.47**	**1.47**	**1.47**	**1.47**	**1.47**	**1.47**	**1.47**	**1.50**
	AAC	0.40	0.40	0.40	0.40	0.53	0.53	0.53	0.53	0.53	0.53	0.53	0.50
Lys	AAA	**1.47**	**1.73**	**1.47**	**1.47**	**1.73**	**1.73**	**1.73**	**1.73**	**1.73**	**1.73**	**1.73**	**1.17**
	AAG	0.53	0.27	0.53	0.53	0.27	0.27	0.27	0.27	0.27	0.27	0.27	0.83
Asp	GAU	0.00-	**1.43@**	0.00-	0.00-	**1.43**	**1.43**	**1.43**	**1.43**	**1.43**	**1.43**	**1.43**	0.80
	GAC	**2.00@**	0.57	2.00 *	2.00 *	0.57	0.57	0.57	0.57	0.57	0.57	0.57	1.20 *
Glu	GAA	**1.82**	**1.17**	**1.82**	**1.82**	**1.17**	**1.17**	**1.17**	**1.17**	**1.17**	**1.17**	**1.17**	**1.75**
	GAG	0.18	0.83	0.18	0.18	0.83	0.83	0.83	0.83	0.83	0.83	0.83	0.25
Ser	UCU	0.86	**2.80**	**1.09**	**1.09**	2.80 *	2.80 *	2.80 *	2.80 *	2.80 *	2.80 *	2.80 *	0.29
	UCC	**1.43**	0.40	**1.36**	**1.36**	0.40	0.40	0.40	0.40	0.40	0.40	0.40	0.86
	UCA	**1.14**	0.40	**1.09**	0.82	0.40	0.40	0.40	0.40	0.40	0.40	0.40	**2.00**
	UCG	0.86	0.80	0.82	**1.09**	0.80	0.80	0.80	0.80	0.80	0.80	0.80	0.86
	AGU	**1.43**	**1.60**	**1.36**	**1.36**	**1.60**	**1.60**	**1.60**	**1.60**	**1.60**	**1.60**	**1.60**	**1.71**
	AGC	0.29	0.00-	0.27	0.27	0.00-	0.00-	0.00-	0.00-	0.00-	0.00-	0.00-	0.29
Pro	CCU	**1.23**	**1.71**	**1.23**	**1.23**	**1.71**	**1.71**	**1.71**	**1.71**	**1.71**	**1.71**	**1.71**	0.80
	CCC	0.62	**1.14**	0.62	0.62	**1.14**	**1.14**	**1.14**	**1.14**	**1.14**	**1.14**	**1.14**	0.00-
	CCA	**1.23**	0.00-	**1.23**	**1.23**	0.00-	0.00-	0.00-	0.00-	0.00-	0.00-	0.00-	**1.60**
	CCG	0.92	**1.14**	0.92	0.92	**1.14**	**1.14**	**1.14**	**1.14**	**1.14**	**1.14**	**1.14**	**1.60**
Thr	ACU	0.40	**2.15@**	0.40	0.40	**2.15**	**2.15**	**2.15**	**2.15**	**2.15**	**2.15**	**2.15**	0.67
	ACC	0.80	**1.54**	0.80	0.80	**1.54**	**1.54**	**1.54**	**1.54**	**1.54**	**1.54**	**1.54**	0.67
	ACA	2.80 *	0.31	2.80 *	2.80 *	0.31	0.31	0.31	0.31	0.31	0.31	0.31	2.67 *
	ACG	0.00-	0.00-	0.00-	0.00-	0.00-	0.00-	0.00-	0.00-	0.00-	0.00-	0.00-	0.00-
Ala	GCU	**1.41**	**3.00@**	**1.18**	**1.18**	**3.00@**	**3.00@**	**3.00@**	**3.00@**	**3.00@**	**3.00@**	**3.00@**	**1.07**
	GCC	0.00-	0.50	0.24	0.24	0.50	0.50	0.50	0.50	0.50	0.50	0.50	**1.07**
	GCA	2.59 *	0.50	2.59 *	2.59 *	0.50	0.50	0.50	0.50	0.50	0.50	0.50	1.87 *
	GCG	0.00-	0.00-	0.00-	0.00-	0.00-	0.00-	0.00-	0.00-	0.00-	0.00-	0.00-	0.00-
Cys	UGU	**2.00**	**2.00**	**2.00**	**2.00**	**2.00**	**2.00**	**2.00**	**2.00**	**2.00**	**2.00**	**2.00**	**2.00**
	UGC	0.00-	0.00-	0.00-	0.00-	0.00-	0.00-	0.00-	0.00-	0.00-	0.00-	0.00-	0.00-
Trp	UGG	1.00	1.00	1.00	1.00	1.00	1.00	1.00	1.00	1.00	1.00	1.00	1.00
Arg	CGU	**1.59**	0.63	**1.45**	**1.45**	0.63	0.63	0.63	0.63	0.63	0.63	0.63	**1.80**
	CGC	0.18	0.00-	0.18	0.18	0.00-	0.00-	0.00-	0.00-	0.00-	0.00-	0.00-	0.30
	CGA	**1.41**	**3.16@**	**1.64**	**1.64**	**3.16@**	**3.16@**	**3.16@**	**3.16@**	**3.16@**	**3.16@**	**3.16@**	**1.20**
	CGG	0.35	0.00-	0.36	0.36	0.00-	0.00-	0.00-	0.00-	0.00-	0.00-	0.00-	0.30
	AGA	**1.76**	**1.42**	**1.45**	**1.45**	**1.42**	**1.42**	**1.42**	**1.42**	**1.42**	**1.42**	**1.42**	**1.20**
	AGG	0.71	0.79	0.91	0.91	0.79	0.79	0.79	0.79	0.79	0.79	0.79	**1.20**
Gly	GGU	**1.11**	**1.85**	**1.11**	**1.11**	**1.85**	**1.85**	**1.85**	**1.85**	**1.85**	**1.85**	**1.85**	0.44
	GGC	0.44	0.31	0.67	0.67	0.31	0.31	0.31	0.31	0.31	0.31	0.31	0.89
	GGA	**2.22**	**1.23**	**2.00**	**2.00**	**1.23**	**1.23**	**1.23**	**1.23**	**1.23**	**1.23**	**1.23**	**2.67**
	GGG	0.22	0.62	0.22	0.22	0.62	0.62	0.62	0.62	0.62	0.62	0.62	0.00-

**Table 4:** Correlation coefficients between the four axes (generated using the correspondence-analysis) and codon usage indices in three gene categories (Photo-, Genet-, and Other-genes) of *Solanum* chloroplast genomes. For Photo-genes, Axis1 to Axis4 accounted for 13.07%, 11.29%, 8.47%, and 8.12%, respectively, of their total variation. For Genet-genes, Axis1 to Axis4 accounted for 16.31%, 13.32%, 11.72%, and 10.34%, respectively, of their total variation. For Other genes, Axis1 to Axis4 accounted for 46.26%, 31.35%, 12.51%, and 4.58%, respectively, of their total variation.

	Photo-Genes				Genet-Genes				Other Genes			
	Axis1	Axis2	Axis3	Axis4	Axis1	Axis2	Axis3	Axis4	Axis1	Axis2	Axis3	Axis4
GC_cds_	0.699 *	0.238 *	0.154 *	0.171 *	0.173 *	−0.058	0.083	0.026	−0.346 *	−0.099	−0.724 *	0.280 @
GC_3s_	0.629 *	−0.127 @	0.151 *	0.083	−0.030	−0.589 *	−0.288 *	0.010	0.419 *	0.644 *	−0.760 *	0.133
Length_cds_	0.170 *	0.169 *	0.294 *	0.167 *	0.047	−0.058	−0.563 *	−0.094	0.404 *	0.720 *	−0.418 *	−0.524 *
ENc	0.254 *	−0.401 *	0.001	−0.143 *	−0.154 @	−0.151 @	−0.520 *	−0.066	0.314 *	0.450 *	−0.163	0.547 *
CAI	0.191 *	0.545 *	−0.043	−0.206 *	0.262 *	0.459 *	−0.235 *	0.287 *	−0.037	−0.609 *	0.791 *	−0.022
Gravy	−0.193 *	0.154 *	0.182 *	−0.193 *	0.016	−0.111	−0.211 *	0.043	0.091	−0.450 *	−0.024	0.771 *
Aromo	0.179 *	−0.226 *	0.449 *	−0.201 *	0.112	−0.111	−0.248 *	−0.238 *	0.485 *	0.158	0.559 *	0.091

GC_cds_: GC content of the coding sequences (>300 bp). GC_3s_: GC content at the synonymous third codon position. Length_cds_: length of the coding sequences (>300 bp). ENc: effective number of codons. CAI: codon adaptation index. Gravy: amino acid hydrophobicity. Aromo: amino acid aromaticity. *: highly significant (*p* < 0.01) and @: significant (*p* < 0.05) using Spearman correlation analysis.

## Supplementary Materials

The following are available online at http://www.mdpi.com/1422-0067/19/10/3142/s1.

## References

[1] Frodin D.G. (2004). History and concepts of big plant genera. Taxon.

[2] Weese T.L., Bohs L. (2007). A three-gene phylogeny of the genus Solanum (Solanaceae). Syst. Bot..

[3] Gargano D., Vezzi A., Scotti N., Gray J., Valle G., Grillo S., Cardi T. The Complete Nucleotide Sequence Genome of Potato (*Solanum tuberosum* cv. Désirée) Chloroplast DNA. https://www.ncbi.nlm.nih.gov/nuccore/NC_008096.

[4] Daniell H., Lee S.-B., Grevich J., Saski C., Quesada-Vargas T., Guda C., Tomkins J., Jansen R.K. (2006). Complete chloroplast genome sequences of *Solanum bulbocastanum*, *Solanum lycopersicum* and comparative analyses with other Solanaceae genomes. Theor. Appl. Genet..

[5] Wu Z. (2016). The completed eight chloroplast genomes of tomato from Solanum genus. Mit DNA.

[6] Cho K.S., Cheon K.S., Hong S.Y., Cho J.H., Im J.S., Mekapogu M., Yu Y.S., Park T.H. (2016). Complete chloroplast genome sequences of *Solanum commersonii* and its application to chloroplast genotype in somatic hybrids with *Solanum tuberosum*. Plant Cell Rep..

[7] Cho K.S., Park T.H. (2016). Complete chloroplast genome sequence of *Solanum nigrum* and development of markers for the discrimination of *S. nigrum*. Hort. Environ. Biotechnol..

[8] Grantham R., Gautier C., Gouy M. (1980). Codon frequencies in 119 individual genes confirm corsistent choices of degenerate bases according to genome type. Nucleic Acids Res..

[9] Osawa S., Ohama T., Yamao F., Muto A., Jukes T.H., Ozeki H., Umesono K. (1988). Directional mutation pressure and transfer RNA in choice of the third nucleotide of synonymous two-codon sets. Proc. Natl. Acad. Sci. USA.

[10] Mackiewicz P., Gierlik A., Kowalczuk M., Dudek M.R., Cebrat S. (1999). How does replication-associated mutational pressure influence amino acid composition of proteins?. Gen. Res..

[11] Mackiewicz P., Kowalczuk M., Mackiewicz D., Nowicka A., Dudkiewicz M., Łaszkiewicz A., Dudek M.R., Cebrat S. (2002). Replication associated mutational pressure generating long-range correlation in DNA. Phys. A.

[12] Sharp P.M., Li W.-H. (1986). Codon usage in regulatory genes in *Escherichia coli* does not reflect selection for “rare” codons. Nucleic Acids Res..

[13] Akashi H. (1994). Synonymous codon usage in *Drosophila melanogaster*: Natural selection and translational accuracy. Genetics.

[14] Ikemura T. (1981). Correlation between the abundance of *Escherichia coli* transfer RNAs and the occurrence of the respective codons in its protein genes: A proposal for a synonymous codon choice that is optimal for the *E. coli* translational system. J. Mol. Biol..

[15] Kanaya S., Yamada Y., Kudo Y., Ikemura T. (1999). Studies of codon usage and tRNA genes of 18 unicellular organisms and quantification of *Bacillus subtilis* tRNAs: Gene expression level and species-specific diversity of codon usage based on multivariate analysis. Gene.

[16] Chiapello H., Lisacek F., Caboche M., Hénaut A. (1998). Codon usage and gene function are related in sequences of *Arabidopsis thaliana*. Gene.

[17] Moriyama E.N., Powell J.R. (1998). Gene length and codon usage bias in *Drosophila melanogaster*, *Saccharomyces cerevisiae* and *Escherichia coli*. Nucleic Acids Res..

[18] Duret L., Mouchiroud D. (1999). Expression pattern and, surprisingly, gene length shape codon usage in Caenorhabditis, Drosophila, and Arabidopsis. Proc. Natl. Acad. Sci. USA.

[19] Orešič M., Shalloway D. (1998). Specific correlations between relative synonymous codon usage and protein secondary structure. J. Mol. Biol..

[20] Romero H., Zavala A., Musto H. (2000). Codon usage in *Chlamydia trachomatis* is the result of strand-specific mutational biases and a complex pattern of selective forces. Nucleic Acids Res..

[21] Zhou T., Weems M., Wilke C.O. (2009). Translationally optimal codons associate with structurally sensitive sites in proteins. Mol. Biol. Evol..

[22] Sau K., Deb A. (2009). Temperature influences synonymous codon and amino acid usage biases in the phages infecting extremely thermophilic prokaryotes. In Silico Biol..

[23] Błażej P., Mackiewicz D., Wnętrzak M., Mackiewicz P. (2017). The impact of selection at the amino acid level on the usage of synonymous codons. G3 Genes Genet..

[24] Chen S.L., Lee W., Hottes A.K., Shapiro L., Mcadams H.H. (2004). Codon usage between genomes is constrained by genome-wide mutational processes. Proc. Natl. Acad. Sci. USA.

[25] Fedorov A., Saxonov S., Gilbert W. (2002). Regularities of context-dependent codon bias in eukaryotic genes. Nucleic Acids Res..

[26] Morton B.R. (2003). The role of context-dependent mutations in generating compositional and codon usage bias in grass chloroplast DNA. J. Mol. Evol..

[27] Plotkin J.B., Kudla G. (2011). Synonymous but not the same: The causes and consequences of codon bias. Nat. Rev. Genet..

[28] Suzuki H., Morton B.R. (2016). Codon adaptation of plastid genes. PLoS ONE.

[29] Shokri E. (2014). Codon bias patterns in photosynthetic genes of halophytic grass *Aeluropus littoralis*. J. Plant Mol. Breed..

[30] Chen X., Cai X., Chen Q., Zhou H., Cai Y., Ben A. (2011). Factors affecting synonymous codon usage bias in chloroplast genome of Oncidium Gower Ramsey. Evol. Bioinform..

[31] Leister D. (2003). Chloroplast research in the genomic age. Trends Genet..

[32] Rosenberg M.S., Subramanian S., Kumar S. (2003). Patterns of transitional mutation biases within and among mammalian genomes. Mol. Biol. Evol..

[33] Willey D.L., Gray J.C. (1990). An open reading frame encoding a putative haem-binding polypeptide is cotranscribed with the pea chloroplast gene for apocytochrome f. Plant Mol. Biol..

[34] Maurizi M.R., Clark W.P., Katayama Y., Rudikoff S., Pumphrey J., Bowers B., Gottesman S. (1990). Sequence and structure of Clp P, the proteolytic component of the ATP-dependent Clp protease of *Escherichia coli*. J. Biol. Chem..

[35] Sueoka N. (1988). Directional mutation pressure and neutral molecular evolution. Proc. Natl. Acad. Sci. USA.

[36] Liu Q. (2006). Analysis of codon usage pattern in the radioresistant bacterium *Deinococcus radiodurans*. Biosystems.

[37] Greenacre M.J. (1984). Theory and Applications of Correspondence Analysis.

[38] Wright F. (1990). The ‘effective number of codons’ used in a gene. Gene.

[39] Sueoka N. (1999). Translation-coupled violation of parity rule 2 in human genes is not the cause of heterogeneity of the DNA G+C content of third codon position. Gene.

[40] Sueoka N. (1995). Intrastrand parity rules of DNA base composition and usage biases of synonymous codons. J. Mol. Evol..

[41] Sharp P.M., Cowe E., Higgins D.G., Shields D.C., Wolfe K.H., Wright F. (1988). Codon usage patterns in *Escherichia coli*, *Bacillus subtilis*, *Saccharomyces cerevisiae*, *Schizosaccharomyces pombe*, *Drosophila melanogaster* and *Homo sapiens*; a review of the considerable within-species diversity. Nucleic Acids Res..

[42] Palmer J.D. (1991). Plastid chromosomes: Structure and evolution. The Molecular Biology of Plastids.

[43] Raubeson L.A., Jansen R.K. (2005). Chloroplast genomes of plants. Diversity and Evolution of Plants-Genotypic and Phenotypic Variation in Higher Plants.

[44] Ravi V., Khurana J.P., Tyagi A.K., Khurana P. (2008). An update on chloroplast genomes. Plant Syst. Evol..

[45] Hershberg R., Petrov D.A. (2009). General rules for optimal codon choice. PLoS Genet..

[46] Wolfe K.H., Sharp P.M. (1988). Identification of functional open reading frames in chloroplast genomes. Gene.

[47] Morton B.R. (1993). Chloroplast DNA codon use: Evidence for selection at the *psbA* locus based on tRNA availability. J. Mol. Evol..

[48] Zhou M., Long W., Li X. (2008). Patterns of synonymous codon usage bias in chloroplast genomes of seed plants. For. Stud. China.

[49] Hershberg R., Petrov D.A. (2010). Evidence that mutation is universally biased towards at in bacteria. PLoS Genet..

[50] Raven J.A., Allen J.F. (2003). Genomics and chloroplast evolution: What did cyanobacteria do for plants?. Genome Biol..

[51] Campbell W.H., Gowri G. (1990). Codon usage in higher plants, green algae, and cyanobacteria. Plant. Physiol..

[52] Kawabe A., Miyashita N.T. (2003). Patterns of codon usage bias in three dicot and four monocot plant species. Genes Genet. Syst..

[53] Chargaff E. (1950). Chemical specificity of nucleic acids and mechanism of their enzymatic degradation. Experientia.

[54] Tillier E.R.M., Collins R.A. (2000). The contributions of replication orientation, gene direction, and signal sequences to base-composition asymmetries in bacterial genomes. J. Mol. Evol..

[55] Charneski C.A., Honti F., Bryant J.M., Hurst L.D., Feil E.J. (2011). Atypical at skew in firmicute genomes results from selection and not from mutation. PLoS Genet..

[56] Necşulea A., Lobry J.R. (2007). A new method for assessing the effect of replication on DNA base composition asymmetry. Mol. Biol. Evol..

[57] Nie X., Deng P., Feng K., Liu P., Du X., You F.M., Song W. (2014). Comparative analysis of codon usage patterns in chloroplast genomes of the Asteraceae family. Plant. Mol. Biol. Rep..

[58] Liu Q., Xue Q. (2005). Comparative studies on codon usage pattern of chloroplasts and their host nuclear genes in four plant species. J. Genet..

[59] Nair R.R., Nandhini M.B., Elango M., Kavitha M., Thilaga S., Sangeetha N., Rao N.S.P., Doss G. (2012). Synonymous codon usage in chloroplast genome of *Coffea arabica*. Bioinformation.

[60] Park T.H. (2017). The complete chloroplast genome of *Solanum berthaultii*, one of the potato wild relative species. Mit DNA Part B.

[61] Sharp P.M., Li W.H. (1987). The codon adaptation index-a measure of directional synonymous codon usage bias, and its potential applications. Nucleic Acids Res..

[62] Karlin S., Mrázek J., Campbell A., Kaiser D. (2001). Characterizations of highly expressed genes of four fast-growing bacteria. J. Bacteriol..

[63] Li G., Wang R., Zhang C., Wang S., He W., Zhang J., Liu J., Cai Y., Zhou J., Su S. (2018). Genetic and evolutionary analysis of emerging H3N2 canine influenza virus. Emerg. Microbes Infect..

[64] Ma J., Li X.-Q. (2015). Organellar genome copy number variation and integrity during moderate maturation of roots and leaves of maize seedlings. Curr. Genet..

[65] Li X.-Q., Chetrit P., Mathieu C., Vedel F., De Paepe R., Remy R., Ambard-Bretteville F. (1988). Regeneration of cytoplasmic male sterile protoclones of *Nicotiana sylvestris* with mitochondrial variations. Curr. Genet..

[66] Wang W., Zhang W., Wu Y., Maliga P., Messing J. (2015). RNA editing in chloroplasts of *Spirodela polyrhiza*, an aquatic monocotelydonous species. PLoS ONE.

[67] Lobry J.R. (1996). Asymmetric substitution patterns in the two DNA strands of bacteria. Mol. Biol. Evol..

[68] Yang Z., Nielsen R. (1998). Synonymous and nonsynonymous rate variation in nuclear genes of mammals. J. Mol. Evol..

[69] Nei M., Gojobori T. (1986). Simple methods for estimating the numbers of synonymous and nonsynonymous nucleotide substitutions. Mol. Biol. Evol..

