# Supplementary material for: Differences in Codon Usage Bias between Photosynthesis-Related Genes and Genetic System-Related Genes of Chloroplast Genomes in Cultivated and Wild Solanum Species
Source: Int J Mol Sci. 2018 Oct 12;19(10):3142. doi: 10.3390/ijms19103142 (PMC6213243; doi:10.3390/ijms19103142)
Supplement: Supplementary file 1 [file ijms-19-03142-s001.pdf]

Table S1. The RSCU values of three gene categories of *Solanum* chloroplast genomes. The optimal codons, preferred codons, and rare codons were identified using the RSCU values. The optimal codon (marked with * ($p < 0.01$) and @ ($p < 0.05$)) was defined as a codon whose usage frequency in the high-expression genes was significantly higher than in the low-expression genes using the chi-squared contingency test. The preferred codon (shown in bold) was a codon with the RSCU value of more than 1 (RSCU > 1), whose investigated usage frequency was more than the expected one, whereas the rare codon (marked with -) was a codon with an RSCU of less than 0.1 (RSCU < 0.1), representing a very low usage frequency.

Amino acid	Codon	Photo-genes	Genet-genes	Other genes	Amino acid	Codon	Photo-genes	Genet-genes	Other genes	Amino acid	Codon	Photo-genes	Genet-genes	Other genes
Phe	UUU	**1.70***	**1.71**	0.92	His	CAU	**2.00***	0.00-	**1.60**	Thr	ACU	**1.67**	**2.00***	**1.90***
	UUC	0.30	0.29@	**1.08***		CAC	0.00-	0.00-	0.40		ACC	0.31	**2.00**	0.19
Leu	UUA	**1.60**	**2.50***	**1.15**	Gln	CAA	**1.53**	**2.00***	**1.62**		ACA	**1.71***	0.00-	**1.14**
	UUG	**1.14**	**2.00**	**1.09@**		CAG	0.47	0.00-	0.38@		ACG	0.31	0.00-	0.76
	CUU	**1.94***	1.00	**1.79**	Asn	AAU	**1.51***	**1.60***	**1.58@**	Ala	GCU	**1.84**	**3.00***	**1.64**
	CUC	0.31*	0.50*	0.77		AAC	0.49	0.40	0.42		GCC	**1.18***	1.00*	0.00-
	CUA	0.85	0.00-	0.70	Lys	AAA	0.79	**1.60***	**1.35**		GCA	0.73	0.00-	**2.00**
	CUG	0.16	0.00-	0.51		AAG	**1.21**	0.40	0.65*		GCG	0.26@	0.00-	0.36
	AUU	**1.72@**	**2.25@**	**1.42**	Asp	GAU	**1.77***	**2.00***	**1.18**	Cys	UGU	**2.00**	0.00-	**1.50@**
Ile	AUC	0.57	0.00-	0.92		GAC	0.23	0.00-	0.82@		UGC	0.00-	0.00-	0.50
	AUA	0.71*	0.75	0.65	Glu	GAA	**1.74**	1.00	**1.47**	Trp	UGG	1.00	0.00-	1.00
Met	AUG	1.00	1.00	1.00		GAG	0.26	1.00*	0.53	Arg	CGU	**3.00**	**1.50**	**3.82***
Val	GUU	**2.07**	0.00-	**2.50@**	Ser	UCU	0.77	**1.33***	**2.07**		CGC	0.00-	0.00-	**1.09@**
	GUC	0.18*	0.00-	0.38		UCC	0.42	0.67	0.44		CGA	0.79	**2.25***	0.00-
	GUA	**1.38**	0.00-	**1.13**		UCA	**2.63***	0.67	**1.96**		CGG	0.00-	0.00-	0.00-
	GUG	0.37@	**4.00***	0.00-		UCG	**1.04***	**1.33**	0.33		AGA	**2.21***	**1.88***	**1.09**
Tyr	UAU	**1.21**	**2.00**	**1.77**		AGU	**1.14**	**2.00**	0.87		AGG	0.00-	0.38	0.00-
	UAC	0.79	0.00-	0.23		AGC	0.00-	0.00-	0.33	Gly	GGU	0.94	**1.33***	**1.33**
STOP	UAA	**3.00**	0.00-	0.00-	Pro	CCU	0.00-	**2.00***	0.47		GGC	0.47	0.00-	0.00-
	UAG	0.00-	**3.00**	0.00-		CCC	1.00*	1.00*	**1.65@**		GGA	**2.14***	**1.33**	**1.33**
	UGA	0.00-	0.00-	**3.00**		CCA	**3.00***	0.00-	**1.18**		GGG	0.44	1.33*	1.33
						CCG	0.00-	1.00	0.71@					

Table S2. Correlation coefficients between four axes and codon usage indices in twelve *Solanum* chloroplast genomes. GC_{cds}: GC content in the coding sequences (>300 bp). GC_{3s}: GC content at the synonymous third codon position. $Length_{cds}$: length of the coding sequences (>300 bp). ENc: effective number of codons. CAI: codon adaptation index. Gravy: amino acid hydrophobicity. Aromo: amino acid aromaticity. *: highly significant ($p < 0.01$) and @: significant ($p < 0.05$) using Spearman correlation analysis.

Axis	Indices	Black Nightshade	Potato			Tomato							
			Wild species		Cultivated species	Wild species							Cultivated species
		S. nigrum	*S. bulbocastanum*	*S. commersonii*	*S. tuberosum*	*S. neorickii*	*S. peruvianum*	*S. chilense*	*S. habrochaites*	*S. pimpinellifolium*	*S. galapagense*	*S. cheesmaniae*	*S. lycopersicum*
Axis 1	GC_{cds}	0.085	−0.010	0.003	−0.031	−0.176	0.182	−0.186	0.147	−0.137	0.164	−0.171	−0.168
	GC_{3s}	0.423*	−0.201	0.256	0.194	−0.385*	0.369*	−0.377*	0.369*	−0.371*	0.380*	−0.381*	−0.324@
	$Length_{cds}$	0.068	0.032	−0.029	−0.027	−0.018	−0.010	0.012	0.009	−0.012	0.011	−0.010	0.028
	ENc	0.475*	−0.212	0.332@	0.246	−0.338@	0.329@	−0.336@	0.312@	−0.368*	0.359*	−0.357*	−0.285@
	CAI	−0.478*	0.404*	−0.422*	−0.399*	0.421*	−0.392*	0.397*	−0.400*	0.480*	−0.457*	0.445*	0.447*
	Gravy	−0.107	0.111	−0.113	−0.113	0.131	−0.127	0.127	−0.137	0.142	−0.156	0.153	0.161
	Aromo	0.072	−0.016	0.030	0.023	−0.020	0.031	−0.029	−0.002	−0.010	−0.006	−0.002	0.027
Axis 2	GC_{cds}	0.125	0.352*	0.406*	−0.358*	0.428*	0.418*	−0.436*	0.462*	−0.438*	−0.430*	−0.427*	0.398*
	GC_{3s}	−0.099	0.155	0.274@	−0.210	0.245	0.329@	−0.319@	0.300@	−0.289@	−0.287@	−0.290@	0.271@
	$Length_{cds}$	−0.276@	−0.113	0.064	0.059	0.168	0.268@	−0.253	0.095	−0.131	−0.138	−0.144	−0.018
	ENc	−0.040	0.067	0.228	−0.129	0.199	0.214	−0.196	0.146	−0.182	−0.180	−0.173	0.160
	CAI	0.192	0.112	0.131	−0.083	0.249	0.177	−0.170	0.067	−0.134	−0.152	−0.133	0.077
	Gravy	−0.097	−0.099	−0.018	0.122	0.142	0.164	−0.153	−0.030	−0.063	−0.062	−0.059	−0.050

	Aromo	0.032	−0.026	0.083	0.012	0.136	0.120	−0.100	0.073	−0.139	−0.138	−0.132	0.029
Axis 3	GC$_{cds}$	−0.385*	0.409*	−0.127	−0.351*	−0.048	−0.290@	−0.295@	−0.099	−0.040	0.060	0.079	0.225
	GC$_{3s}$	−0.305@	0.498*	−0.375*	−0.422*	0.238	0.108	0.105	−0.224	0.229	−0.228	−0.222	0.301@
	Length$_{cds}$	−0.265@	0.408*	−0.374*	−0.381*	0.368*	0.285@	0.301@	−0.278@	0.376*	−0.389*	−0.386*	0.325@
	ENc	−0.170	0.263@	−0.201	−0.220	0.013	0.022	0.030	−0.187	0.028	−0.046	−0.048	0.123
	CAI	−0.243	0.053	0.154	−0.105	−0.186	−0.303@	−0.286@	−0.212	−0.214	0.173	0.199	0.014
	Gravy	−0.124	0.063	−0.021	−0.093	0.076	0.069	0.075	−0.273@	0.036	−0.071	−0.058	0.060
	Aromo	−0.139	0.161	−0.073	−0.181	0.035	0.049	0.054	−0.240	0.033	−0.058	−0.056	0.093
Axis 4	GC$_{cds}$	0.195	−0.034	−0.084	0.029	0.038	−0.123	−0.144	−0.166	−0.049	0.041	−0.048	0.134
	GC$_{3s}$	−0.018	0.019	0.079	−0.128	−0.088	0.024	0.029	0.024	−0.167	0.135	−0.137	0.025
	Length$_{cds}$	−0.099	0.155	0.109	−0.180	−0.071	0.069	0.099	0.236	−0.118	0.117	−0.113	−0.041
	ENc	−0.162	−0.030	0.194	−0.126	−0.052	0.053	0.108	−0.055	−0.101	0.081	−0.067	0.122
	CAI	−0.030	−0.280@	−0.368*	0.475*	−0.212	0.202	0.208	−0.351*	−0.264@	0.308@	−0.314@	0.415*
	Gravy	0.020	−0.080	−0.185	0.200	−0.232	0.191	0.187	−0.077	−0.257	0.269@	−0.282@	0.174
	Aromo	−0.168	−0.114	−0.103	0.123	−0.228	0.213	0.234	−0.076	−0.252	0.272@	−0.265@	0.185

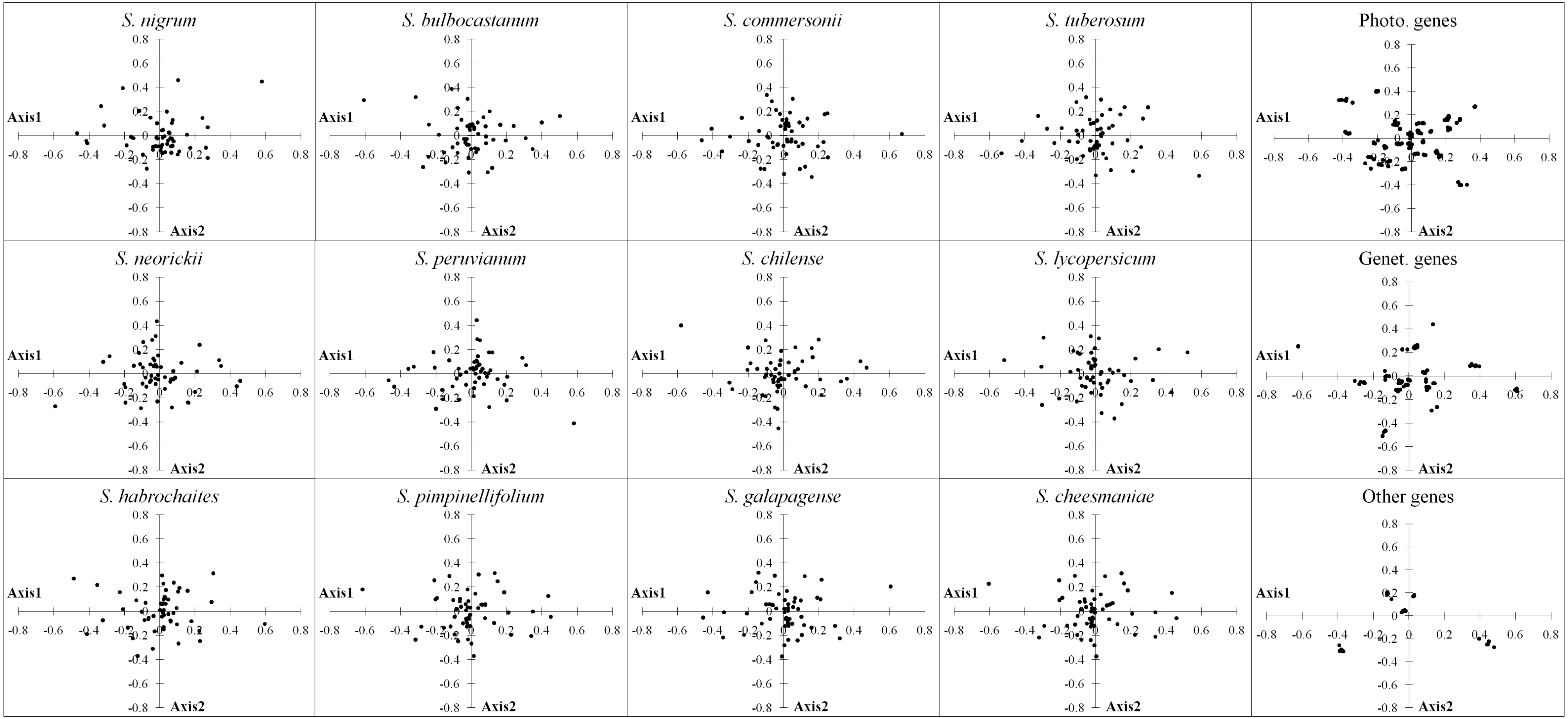

Figure S1. The correspondence analysis (COA) of the genes in *Solanum* chloroplast genomes. Each point represents a gene corresponding to the coordinates of the first (Axis1) and second (Axis2) axes of variation generated from the correspondence analysis. For twelve chloroplast genomes of *S. nigrum*, *S.*

bulbocastanum, S. commersonii, S. tuberosum, S. neorickii, S. peruvianum, S. chilense, S. lycopersicum, S. habrochaites, S. pimpinellifolium, S. galapagense, S. cheesmaniae, Photo-genes, Genet-genes, and Other genes, Axis1 accounted for 9.96%, 9.89%, 9.56%, 9.61%, 9.91%, 9.86%, 9.87%, 9.93%, 9.84%, 9.93%, 9.91%, 9.89%, 13.07%, 16.31%, and 46.26%, respectively, of their total variation; Axis2 accounted for 7.71%, 7.93%, 7.76%, 7.76%, 7.74%, 7.60%, 7.60%, 7.84%, 7.81%, 7.75%, 7.82%, 7.79%, 11.29%, 13.32%, and 31.35%, respectively, of total variation; Axis3 accounted for 7.30%, 7.28%, 7.37%, 7.49%, 7.42%, 7.37%, 7.37%, 7.41%, 7.54%, 7.41%, 7.40%, 7.40%, 8.47%, 11.72%, and 12.51%, respectively, of their total variation; and Axis4 accounted for 6.78%, 6.97%, 6.97%, 6.90%, 6.95%, 6.85%, 6.86%, 7.03%, 7.28%, 7.07%, 7.04%, 7.03%, 8.12%, 10.34%, and 4.58%, respectively, of their total variation. The first four axes jointly accounted for 31.94%, 32.07%, 31.59%, 31.81%, 31.92%, 31.69%, 31.87%, 32.46%, 32.26%, 32.13%, 32.16%, 33.20%, 43.17%, 45.93%, and 90.12%, respectively, of the total variation. Thus, the first four axes can be considered the main factors that affected the codon usage bias in *Solanum* chloroplast genomes.
